# Worldwide audit of blood transfusion practice in critically ill patients

**DOI:** 10.1186/s13054-018-2018-9

**Published:** 2018-04-19

**Authors:** Jean-Louis Vincent, Ulrich Jaschinski, Xavier Wittebole, Jean-Yves Lefrant, Stephan M. Jakob, Ghaleb A. Almekhlafi, Tommaso Pellis, Swagata Tripathy, Paolo N. Rubatto Birri, Yasser Sakr, on behalf of the ICON Investigators

**Affiliations:** 1 Department of Intensive Care, Erasme University Hospital, Unversité Libre de Bruxelles, Route de Lennik 808, 1070 Brussels, Belgium; 2 Klinik für Anästhesiologie und operative Intensivmedizin, Klinikum Augsburg, Augsburg, Germany; 3 Critical Care Department, Cliniques universitaires St Luc, UCL, Brussels, Belgium; 4 Service des Réanimations, Division Anesthésie Réanimation Douleur Urgence, CHU Nîmes, Nîmes, France; 5 Department of Intensive Care Medicine, University Hospital Bern, University of Bern, Bern, Switzerland; 6 ICS Department, Prince Sultan Military Medical City, Riyadh, Saudi Arabia; 7 Anesthesia and Intensive Care, Santa Maria degli Angeli Hospital, Pordenone, Italy; 8 Department of Anesthesia, AIIMS, Sijua, Patrapada, Bhubaneswar, Odisha India; 9 Department of Anesthesiology and Intensive Care, Universitätsklinikum Jena, Jena, Germany

**Keywords:** Red blood cell, Worldwide, Severity of illness

## Abstract

**Background:**

The aim was to describe transfusion practice in critically ill patients at an international level and evaluate the effects of red blood cell (RBC) transfusion on outcomes in these patients.

**Methods:**

This was a pre-planned sub-study of the Intensive Care Over Nations audit, which involved 730 ICUs in 84 countries and included all adult patients admitted between 8 May and 18 May 2012, except admissions for routine postoperative surveillance.

**Results:**

ICU and hospital outcomes were recorded. Among the 10,069 patients included in the audit, data related to transfusion had been completed for 9553 (mean age 60 ± 18 years, 60% male); 2511 (26.3%) of these had received a transfusion, with considerable variation among geographic regions. The mean lowest hemoglobin on the day of transfusion was 8.3 ± 1.7 g/dL, but varied from 7.8 ± 1.4 g/dL in the Middle East to 8.9 ± 1.9 g/dL in Eastern Europe. Hospital mortality rates were higher in transfused than in non-transfused patients (30.0% vs. 19.6%, *p* < 0.001) and increased with increasing numbers of transfused units. In an extended Cox proportional hazard analysis, the relative risk of in-hospital death was slightly lower after transfusion in the whole cohort (hazard ratio 0.98, confidence interval 0.96–1.00, *p* = 0.048). There was a stepwise decrease in the hazard ratio for mortality after transfusion with increasing admission severity scores.

**Conclusions:**

More than one fourth of critically ill patients are transfused during their ICU stay, with considerable variations in transfusion practice among geographic regions. After adjustment for confounders, RBC transfusions were associated with a slightly lower relative risk of in-hospital death, especially in the most severely ill patients, highlighting the importance of taking the severity of illness into account when making transfusion decisions.

**Electronic supplementary material:**

The online version of this article (10.1186/s13054-018-2018-9) contains supplementary material, which is available to authorized users.

## Background

Red blood cell (RBC) transfusions are frequently administered in critically ill patients [1, 2], although probably less so in recent years than in the past. RBC transfusion may have beneficial effects by increasing oxygen delivery to the tissues and improving the oxygen demand/supply balance [3], but may also have some harmful effects. In addition to possible type and cross-matching issues, transfusions may be associated with transfusion-related acute lung injury (TRALI), transfusion-associated circulatory overload (TACO) and immunomodulating effects resulting in a higher incidence of nosocomial infections [4].

Prospective, randomized, controlled trials (RCTs) have suggested that restrictive RBC transfusion strategies are safe, but this may apply more to patients who are less severely ill. Indeed, recent meta-analyses have suggested caution in patients with cardiovascular disease [5], and that restrictive strategies should be applied with particular care in high-risk patients undergoing major surgery [6]. The uncertainty concerning the risk/benefit profile of RBC transfusion in critically ill patients has fueled ongoing debate on the subject over the last two decades. Moreover, the heterogeneity of critical illness and the absence of universally accepted guidelines may influence RBC transfusion practice in intensive care units (ICUs) worldwide. Several regional observational studies [1, 7] have reported on patterns of RBC transfusion in ICU patients, but global differences in transfusion practice have not been addressed. Such data would be useful from an epidemiological point of view, and also to improve our understanding of the factors that may influence transfusion practice.

We used the worldwide Intensive Care Over Nations (ICON) database, to investigate current transfusion practices worldwide and the possible effect of transfusion on outcomes.

## Methods

This was a planned post-hoc analysis of the ICON database. ICON was a multicenter, worldwide audit endorsed by the World Federation of Societies of Intensive Care and Critical Care Medicine (WFSICCM). Institutional recruitment for participation was by open invitation and was voluntary, with no financial incentive. Institutional review board approval was obtained by the participating institutions according to local ethical regulations. Informed consent was not required due to the observational and anonymous nature of data collection in this audit. The ICON audit collected data on 10,069 adult (> 16 years of age) patients admitted to the participating centers (Additional file 1) between 8 May and 18 May 2012. The 9553 patients for whom data related to transfusion had been completed were included in the present analysis.

### Data collection and management

Details about the conduct of the audit, the data collected and the definitions used are provided elsewhere [8]. Data were collected prospectively using preprinted case report forms (CRFs) and were electronically introduced by the investigators using an Internet-based website. Data collection on admission included demographic data and data on comorbid diseases (chronic obstructive pulmonary disease (COPD), cancer, metastatic cancer, hematologic cancer, insulin-dependent diabetes mellitus, heart failure (New York Heart Association (NYHA) class III-IV), chronic renal failure, human immunodeficiency virus (HIV) infection, cirrhosis, immunosuppression, steroid therapy or chemotherapy). Clinical data and laboratory data were collected daily for a maximum of 28 days in the ICU, and included respiratory, hemodynamic and hematological parameters, and fluid administration. For the purposes of this study, the minimum hemoglobin concentration reported on the day of the transfusion was considered as the “transfusion trigger”. ICU and hospital length of stay and outcomes were recorded. Clinical and laboratory data for the Simplified Acute Physiology Score (SAPS) II [9] were reported as the worst value within 24 h after admission. A daily evaluation of organ function according to the sequential organ failure assessment (SOFA) score [10] was performed and organ failure was defined as a SOFA score >2 for the organ in question. Surgical admissions were identified as patients who had had surgery in the 4 weeks preceding admission.

Data were processed and analyzed in the Department of Intensive Care of the University of Brussels. The study coordinators processed all queries during data collection. Detailed instructions, explaining the aim of the study, instructions for data collection, and definitions for various items were available through a secured website for all participants. Validity checks were made concurrent with data entry by the electronic case record form, including plausibility checks within each variable and between variables. Data were further reviewed by the coordinating center for plausibility and availability of the outcome parameter, and doubts were clarified with the corresponding ICU. We did not perform any other supplementary quality control measures.

### Subgroup analyses

Subgroups defined a priori included male versus female patients, medical versus surgical patients, sepsis versus no sepsis, and subgroups according to quartiles of SAPS II and SOFA scores on admission to the ICU and age categories (18–50, 51–65, 66–75 or > 75 years).

### Statistical analysis

Data are summarized using means with standard deviation (SD), medians and interquartile ranges (IQ) or numbers and percentages. For the purposes of this study, hemoglobin concentrations were categorized according to cutoff values of < 7, 7–8.9, 9–10.9, 11–13, and > 13 g/dL. Differences between groups were tested using analysis of variance (ANOVA), the Kruskal-Wallis test, Student’s *t* test, the Mann-Whitney test, the chi-square test or Fisher’s exact test as appropriate.

To determine the adjusted relative risk of hospital death after RBC transfusion during the ICU stay, we developed an extended multivariable Cox model in the overall population and in the subgroups defined a priori. We considered discharge as a competing event. Other confounding variables considered for the Cox analysis included age, sex, SAPS II score and SOFA sub-scores on admission to the ICU, type of admission, the use of mechanical ventilation or renal replacement therapy on ICU admission, comorbidities, presence of sepsis and hemoglobin categories on admission to the ICU. We also adjusted for ICU and hospital-related organizational factors including type of hospital, hospital bed capacity, total number of ICU patients in 2011, number of staffed ICU beds, number of nurses and the presence of 24 h/day in-house intensivist coverage. The geographic region (North America, South America, Western Europe, Eastern Europe, Middle East, South Asia, East and South-East Asia, Oceania and Africa) was considered in these multivariable models. An extended Cox model was constructed, adding interaction terms that involve time, i.e., time-varying fixed effects, computed as the by-product of time and individual covariates in the model (time × covariate). Individual time-varying fixed covariates were introduced one by one and in combinations in the extended model, none of which was found to be significant (Wald chi-square statistics). Colinearity between variables was checked before modeling and all variables were included in the models thereafter, irrespective of the level of significance on univariate basis. Transfusion during the ICU stay was introduced into the model as a time-dependent variable using the time to the first erythrocyte unit given.

Data were analyzed using IBM® SPSS® Statistics software, version 22 for windows. All reported *p* values are two-sided. A *p* value of less than 0.05 was considered to indicate statistical significance.

## Results

### Characteristics of the study group

The characteristics of the 9553 patients (mean age 60 ± 18 years, 60% male) on ICU admission are shown in Table 1; 56% were medical admissions. Median ICU and hospital lengths of stay were 3 (2-6) and 10 (5-20) days, respectively, and the overall ICU and hospital mortality rates were 16.2% and 22.3%, respectively. Patient characteristics and outcomes varied among geographic regions (Additional file 2: Table S1).

**Table 1:** Clinical characteristics and outcomes of the study cohort according to whether or not they received a transfusion during their ICU stay

Characteristic	All patients *N* = 9553	No transfusion *N* = 7042	Transfusion *N* = 2511	*p* value
Age (years), mean ± SD	59.9 ± 18.0	59.5 ± 18.3	60.9 ± 17.3	0.002
Male, *n* (%)	5669 (60.1)	4180 (60.2)	1489 (59.8)	0.736
Admission severity scores, mean ± SD				
SAPS II	41.3 ± 17.7	39.7 ± 17.7	45.7 ± 17.1	<0.001
SOFA score	6.4 ± 4.2	5.4 ± 3.7	8.1 ± 4.3	<0.001
Type of admission, *n* (%)				<0.001
Medical	5089 (56.1)	4097 (61.2)	992 (40.9)	
Surgical	3317 (36.6)	2063 (31.0)	1254 (51.7)	
Trauma	607 (6.7)	436 (6.6)	171 (6.8)	
Others	61 (0.7)	51 (0.8)	10 (0.4)	
Source of admission, *n* (%)				<0.001
Ambulance	3590 (37.6)	2974 (42.2)	616 (24.5)	
Hospital floor	2512 (26.3)	1703 (24.2)	809 (32.2)	
OR/ER	1749 (18.3)	1081 (15.4)	668 (26.6)	
Other hospital	937 (9.8)	674 (9.6)	263 (10.5)	
Others	765 (8.0)	610 (8.7)	155 (6.2)	
Comorbid conditions				
COPD	1182 (12.4)	907 (12.9)	275 (11.0)	0.012
Diabetes mellitus insulin-dependent	925 (9.7)	644 (9.1)	281 (11.2)	0.003
Chronic heart failure (NYHA III-IV)	875 (9.2)	627 (8.9)	248 (9.9)	0.147
Chronic renal failure	875 (9.2)	549 (7.8)	326 (13.0)	<0.001
Non-metastatic cancer	1011 (10.6)	659 (9.4)	352 (14.0)	<0.001
Immunosuppression	727 (7.6)	459 (6.5)	268 (10.7)	<0.001
Cirrhosis	334 (3.5)	183 (2.6)	151 (6.0)	<0.001
Metastatic cancer	320 (3.3)	201 (2.9)	119 (4.7)	<0.001
Hematological cancer	207 (2.2)	110 (1.6)	97 (3.9)	<0.001
HIV	69 (0.7)	46 (0.7)	23 (0.9)	0.182
Number of comorbidities, *n* (%)				<0.001
Any	5195 (54.4)	3012 (42.8)	1346 (53.6)	
1	2667 (27.9)	1936 (27.5)	731 (29.1)	
2	1162 (12.2)	755 (10.7)	407 (16.2)	
3	403 (4.2)	245 (3.5)	158 (6.3)	
≥ 4	126 (1.3)	76 (1.1)	50 (2.0)	
Mortality rates, *n* (%)				
ICU	1507 (16.2)	976 (14.3)	531 (21.5)	<0.001
Hospital	2006 (22.3)	1286 (19.6)	720 (30.0)	<0.001
Length of stay, all patients, median (IQR)				
ICU	3.0 (2.0–6.0)	3.0 (1.0–5.0)	5.0 (3.0–11.0)	<0.001
Hospital	10.0 (5.0–20.0)	8.0 (4.0–16.0)	15.0 (7.0–30.0)	<0.001
Length of stay, survivors, median (IQR)				
ICU	3.0 (2.0–6.0)	2.0 (1.0–6.0)	5.0 (3.0–11.0)	<0.001
Hospital	11.0 (6.0–21.0)	10.0 (5.0–18.0)	17.0 (9.0–35.0)	<0.001
Length of stay, non-survivors, median (IQR)				
ICU	3.0 (1.0–8.0)	2.0 (1.0–6.0)	7.0 (2.5–15.0)	<0.001
Hospital	5.0 (2.0–13.0)	4.0 (1.0–9.0)	9.5 (3.0–20.5)	<0.001

Missing values: age, sex, and type of admission in 50, 114 and 479 patients, respectively; percentages were calculated taking into account missing values

*COPD* chronic obstructive pulmonary disease*, HIV* human immunodeficiency viral infection, *ICU* intensive care unit, *NYHA* New York Heart Association Classification, *SAPS* simplified acute physiology score, *SOFA* sequential organ assessment, *SD* standard deviation, *ER* emergency room, *OR* operating room

### Global patterns of anemia and its relationship with outcome

The mean lowest hemoglobin concentration on the day of ICU admission was 10.7 ± 2.4 g/dL; 26.4% of patients had a hemoglobin concentration < 9 g/dL on admission and 44.1% of patients had a hemoglobin concentration < 9 g/dL during the ICU stay. Hemoglobin concentrations were lower during the first 2 weeks following ICU admission in patients who received a transfusion than in those who did not (Additional file 2: Figure S1). In patients who did not receive a transfusion at any time during the ICU stay, hemoglobin concentrations decreased steadily after admission to the ICU (Additional file 2: Figure S1).

**Fig. 1:**
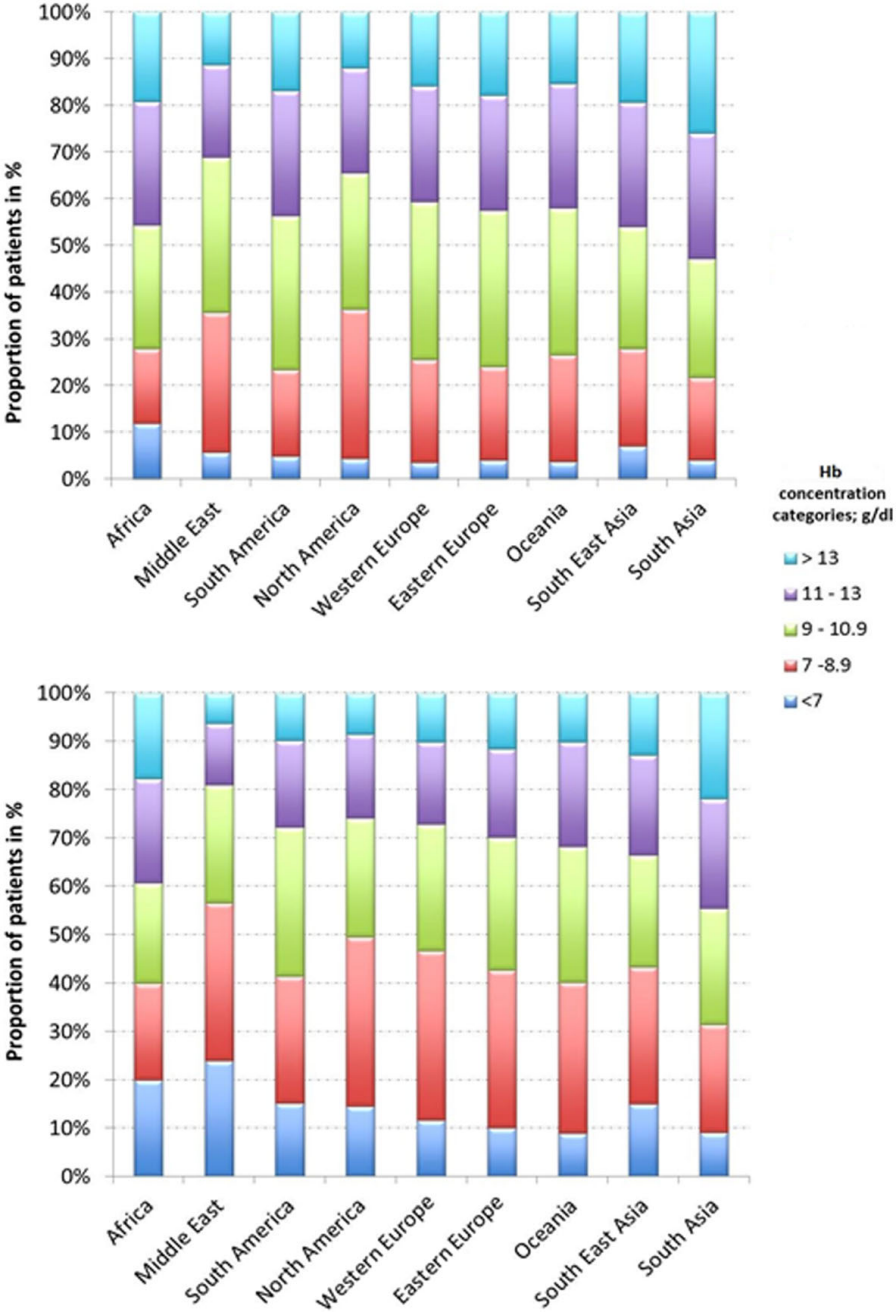
Distribution of hemoglobin concentrations in the different geographic regions on admission (upper panel) and during the ICU stay (lower panel)

Hemoglobin concentrations varied considerably in the different geographic regions (Fig. 1). On the day of ICU admission, mean hemoglobin concentrations varied from 10.1 ± 2.3 g/dL in the Middle East to 11.3 ± 2.5 g/dL in South Asia. The proportion of patients with severe anemia (hemoglobin concentration < 7 g/dL) on admission to the ICU ranged from 3.5% (Western Europe) to 12.0% (Africa). The overall prevalence of anemia (hemoglobin concentration < 9 g/dL) at any time during the ICU stay ranged from 31.5% (South Asia) to 56.6% (Middle East).

There was an inverse relationship between the lowest hemoglobin concentration on the day of ICU admission and during the ICU stay and ICU and hospital mortality rates and the degree of organ dysfunction/failure as assessed by the SOFA score during the ICU stay (Additional file 2: Table S2, Figure S2). Patients with a lowest hemoglobin concentration of < 7 g/dL had an almost twofold higher mortality rate than those with a lowest hemoglobin concentration of > 13 g/dL on the day of ICU admission (Additional file 2: Figure S2) or at any time during the ICU stay. However, differences in mortality rates for hemoglobin concentrations between 7 and 13 g/dL were less marked.

### Transfusion practice

**Fig. 2:**
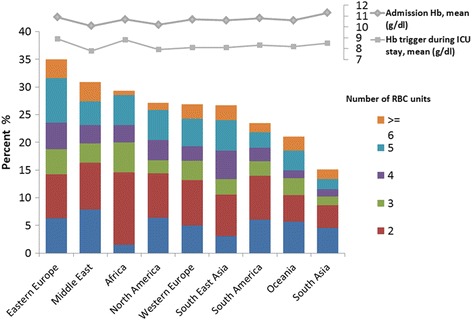
Proportion of patients who received a red blood cell (RBC) transfusion during the ICU stay in the different geographic regions. Hb, hemoglobin

A total of 2511 (26.3%) patients received a RBC transfusion during the ICU stay; 1192 (12.5%) patients received a transfusion on the day of ICU admission. The proportion of patients who received a transfusion varied considerably among the different geographic regions, from 7.1% in South Asia to 19.4% in Eastern Europe on the day of ICU admission, and from 15% in South Asia to 34.9% in Eastern Europe during the ICU stay (Fig. 2; Additional file 2: Table S1).

There were 62% of patients with hemoglobin levels < 7 g/dL who received a RBC transfusion during the ICU stay, whereas only 3.7% of patients with hemoglobin concentrations >13 g/dL received a transfusion (Additional file 2: Table S3). The proportion of patients who received a transfusion during the ICU stay increased with increasing ICU length of stay (Additional file 1: Table S4). Patients who received a transfusion at any time during the ICU stay had higher SAPS II and SOFA scores on admission to the ICU than those who did not (Table 1). The median number of units transfused was 3 (IQ 2–5).

The mean lowest hemoglobin concentration on the day of transfusion (transfusion trigger) for patients transfused on the day of admission to the ICU was 8.3 ± 2.2 g/dL, but varied according to age, type of admission, the presence of sepsis, severity scores, ICU length of stay and geographic region (Table 2). The transfusion trigger was higher in surgical patients than in medical patients for transfusions on the day of admission and at any time during the ICU stay. Hemoglobin concentrations on the day of transfusion were lower in patients with higher scores for severity of illness than in patients with lower scores (Additional file 2: Figure S3). The lowest transfusion trigger on the day of transfusion was reported in the Middle East (7.7 ± 2.3 g/dL on the day of admission to the ICU and 7.8 ± 1.4 g/dL at any time during the ICU stay) and was the highest in Africa on the day of admission (9.0 ± 2.4 g/dL) and in Eastern Europe at any time during the ICU stay (8.9 ± 1.9) (Table 2, Figure 2).

**Table 2:** Characteristics of patients according to the lowest hemoglobin concentration on the day of transfusion

	Lowest hemoglobin concentration on day of transfusion, mean ± SD					
Characteristic	Transfusion on day of admission		Transfusion at any time during ICU stay^a^			
	Number		Number^b^	Min	Max	Mean
All patients	1170	8.3 ± 2.2	2470	7.9 ± 1.7	8.6 ± 1.8	8.3 ± 1.7
Age, years				***	*	***
16–50	312	8.2 ± 2.4	605	7.7 ± 1.9	8.5 ± 1.9	8.1 ± 1.8
51–65	321	8.4 ± 2.1	731	7.9 ± 1.6	8.6 ± 1.7	8.2 ± 1.6
66–75	267	8.5 ± 2.1	578	8.0 ± 1.8	8.7 ± 1.7	8.4 ± 1.6
>75	267	8.3 ± 2.0	551	8.1 ± 1.7	8.7 ± 1.7	8.4 ± 1.7
Sex						
Female	463	8.3 ± 2.1	984	7.9 ± 1.7	8.6 ± 1.8	8.2 ± 1.6
Male	693	8.3 ± 2.2	1466	7.9 ± 1.7	8.6 ± 1.8	8.3 ± 1.6
Type of admission		***		***	***	***
Surgical	671	8.8 ± 2.1	1237	8.2 ± 1.7	8.9 ± 1.8	8.6 ± 1.7
Medical	392	7.6 ± 2.1	973	7.5 ± 1.7	8.3 ± 1.7	7.9 ± 1.6
Trauma	69	8.4 ± 2.1	167	8.0 ± 1.6	8.7 ± 1.7	8.3 ± 1.6
Others	3	6.8 ± 2.6	10	7.9 ± 2.2	8.5 ± 2.2	8.2 ± 2.2
SAPS II		***		***	***	***
0–28	188	8.7 ± 2.4	345	8.2 ± 2.0	8.9 ± 2.0	8.6 ± 1.9
29–39	319	8.7 ± 2.2	657	8.2 ± 1.8	8.8 ± 1.8	8.5 ± 1.7
40–51	284	8.1 ± 2.0	655	7.7 ± 1.5	8.4 ± 1.6	8.1 ± 1.4
52–119	379	8.0 ± 2.0	813	7.7 ± 1.7	8.5 ± 1.8	8.1 ± 1.6
SOFA score on admission to the ICU		*		***		**
0–3	188	8.5 ± 2.5	353	8.2 ± 2.1	8.8 ± 1.9	8.5 ± 1.9
4–5	169	8.5 ± 2.3	375	8.1 ± 1.8	8.7 ± 1.8	8.4 ± 1.8
6–8	292	8.5 ± 2.0	665	8.0 ± 1.6	8.6 ± 1.7	8.3 ± 1.5
>8	521	8.1 ± 2.1	1077	7.7 ± 1.7	8.6 ± 1.8	8.1 ± 1.6
ICU length of stay, days		***		***	***	***
1	197	7.8 ± 2.3	235	7.8 ± 2.2	8.0 ± 2.2	7.9 ± 2.2
2–3	360	8.4 ± 2.1	589	8.2 ± 1.8	8.6 ± 1.8	8.4 ± 1.7
4–7	318	8.5 ± 2.1	629	8.1 ± 1.7	8.7 ± 1.8	8.4 ± 1.7
>7	271	8.3 ± 2.1	944	7.6 ± 1.5	8.7 ± 1.7	8.1 ± 1.4
Sepsis		*		**		
No	734	8.4 ± 2.2	1283	8.1 ± 1.9	8.6 ± 1.8	8.4 ± 1.8
Yes	436	8.1 ± 2.0	1187	7.7 ± 1.6	8.6 ± 1.7	8.1 ± 1.5
Geographic region		***		***	**	***
Africa	24	9.0 ± 2.4	38	8.5 ± 2.2	9.1 ± 2	8.8 ± 2
Middle East	35	7.7 ± 2.3	111	7.4 ± 1.4	8.3 ± 1.6	7.8 ± 1.4
South America	86	8.7 ± 2.4	205	8.0 ± 2.0	8.7 ± 2.0	8.3 ± 1.9
North America	92	8.2 ± 2	183	7.5 ± 1.6	8.3 ± 1.6	7.9 ± 1.4
Western Europe	510	8.1 ± 1.8	1099	7.8 ± 1.4	8.5 ± 1.5	8.1 ± 1.4
Eastern Europe	211	8.8 ± 2.2	378	8.4 ± 2	9.3 ± 2	8.9 ± 1.9
Oceania	39	8.5 ± 1.9	91	7.9 ± 1.5	8.5 ± 1.5	8.2 ± 1.4
South East Asia	110	8.0 ± 2.4	232	7.7 ± 1.9	8.5 ± 1.9	8.1 ± 1.8
South Asia	63	8.8 ± 3.2	133	8.1 ± 2.5	8.9 ± 2.4	8.5 ± 2.3

*Hb* hemoglobin, *SOFA* Sequential Organ Failure Assessment, *SAPS II* Simplified Acute Physiology Score II, *ICU* intensive care unit, *IQ* interquartile

^a^Some patients were transfused several times hence, minimum (Min), maximum (Max) and mean values are given

^b^In total 2511 patients received a transfusion but numbers in different categories vary because of missing data

*p = 0.01–0.05; **p = 0.01–0.001; ***p < 0.001 among groups

### Outcomes according to RBC transfusion status

ICU and hospital mortality rates were higher in patients who received a transfusion during the ICU stay than in those who did not (21.5% vs. 14.3% and 30.0% vs. 19.6%, both *p* < 0.001) (Table 1). Mortality rates increased with increasing numbers of transfused RBC units (Additional file 2: Table S5). In the multivariable Cox analysis in the whole cohort, the relative risk of in-hospital death was slightly lower after transfusion (hazard ratio 0.98, confidence interval 0.96–1.00, *p* = 0.048) (Additional file 2: Table S6).

### Subgroup analysis

**Fig. 3:**
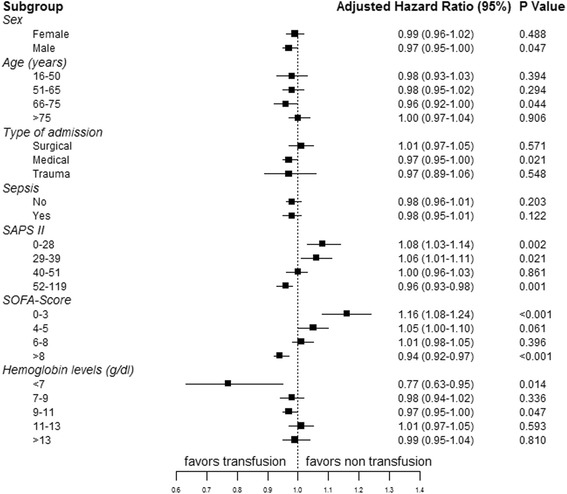
Forest plot was created from modelling of our observational data showing the risk of in-hospital death after transfusion in the various subgroups of patients. SAPS, Simplified Acute Physiology Score; SOFA, Sequential Organ Failure Assessment (on admission)

The adjusted hazard ratios of in-hospital mortality among the pre-defined sub-groups are shown in Fig. 3. Transfusion was associated with a lower hazard of death in medical patients, in patients with a hemoglobin level < 7 g/dL, and in patients with higher SAPS II (52–119) and SOFA scores (>8) on ICU admission. There was a stepwise decrease in hazard ratio for mortality with transfusion according to increasing admission severity scores (Fig. 3).

## Discussion

The present study provides interesting information about the epidemiology and impact of RBC transfusions in ICUs around the globe. Despite the fact that the degree of anemia in our study was comparable to that reported in previous observational studies [1], the proportion of patients who received a RBC transfusion (26%) is lower than that reported in older studies: for example, 37% in the European ABC study [1] and 44% in the North American CRIT study [7], which were comparable studies conducted about 15 years ago, and 33% in the European SOAP study conducted a few years later [2]. The transfusion triggers in our study were slightly lower in patients admitted to ICUs from Western Europe (8.1 g/dL) and North America (7.9 g/dL) than those reported in the older ABC (8.4 g/dL) and CRIT (8.6 g/dL) studies.

We observed considerable variation in transfusion practice among geographic regions, and marked differences in pre-transfusion hemoglobin concentrations according to age, type of admission, the presence of sepsis, severity scores and ICU length of stay. There are surprisingly few data on ICU transfusion practice in areas of the world other than Europe, Oceania and North America.

As expected, transfusions were associated with higher severity scores, longer lengths of stay and higher mortality rates. The important finding was that in multivariable analysis, RBC transfusion was associated with greater benefit in sicker patients and with worse outcomes in patients with lower severity scores. In the study by Hebert et al. [11], mortality rates were also higher in the liberal-strategy group than in the restrictive-strategy group in patients with lower Acute Physiology and Chronic Health Evaluation (APACHE) II scores.

A particular strength of this study is the large size of the database, with participants from all geographical regions; nevertheless, we acknowledge that contributions from some areas were limited and may not be representative of the area in general. We also recognize other limitations. First, contribution to the study was voluntary and inclusion may have been subject to selection bias, particularly in the less-represented regions. Moreover, we did not adjust for repeated measures within ICUs or regions, so that small numbers of clinicians within a few large ICUs contributing a large amount of data may have influenced the estimates, especially in smaller regions. Second, although this was a predefined sub-study, the original ICON study was not specifically designed to investigate this topic and some potentially relevant aspects were not available. For example, the indication for red blood cell transfusion, especially acute bleeding, was not recorded in our database, so that a possible confounding effect cannot be excluded. In addition, availability of blood for transfusion at the regional level and differences in local transfusion guidelines may influence decision making in this context. Nonetheless, we did adjust for several factors related to the severity of illness and the underlying disease process. As an observational study, we cannot control for unmeasured confounding as can RCTs, and can therefore only demonstrate an association and not prove causality; our findings can therefore only be considered as hypothesis-generating. Nevertheless, unlike RCTs in which only a fraction of potentially eligible patients are included, observational studies include all patients thus providing data that are more pragmatic and generalizable to the whole ICU population. Third, although transfusion was associated with a lower hazard of mortality in sicker patients, we are unable to comment on optimum thresholds or targets of transfusion. Fourth, we lack information on complications associated with transfusions, such as TRALI, TACO, transfusion reactions and development of nosocomial infections. We also have no information on whether or not transfused units were leukocyte-depleted.

## Conclusions

Despite the limitations mentioned, this large worldwide study of patients admitted to the ICU shows clear differences in transfusion practice in different geographic regions. Our results also suggest that RBC transfusion may be associated with better outcomes in the most severely ill patients, highlighting the importance of taking severity of illness into account when deciding on the need for transfusion in an individual patient.

## Additional files

Additional file 1: Alphabetical list of participating centers in the Intensive Care Over Nations (ICON) audit, by country. (PDF 162 kb)

Additional file 2: **Tables S1-S6** and **Figures S1-S3**. (PDF 459 kb)

## Abbreviations

COPD: Chronic obstructive pulmonary disease
Hb: Hemoglobin
ICON: Intensive Care Over Nations
ICU: Intensive care unit
NYHA: New York Heart Association
RBC: Red blood cell
RCT: Randomized controlled trial
SAPS: Simplified Acute Physiology Score
SOFA: Sequential Organ Failure Assessment
TACO: Transfusion-associated circulatory overload
TRALI: Transfusion-related acute lung injury
