# Supplementary material for: Worldwide audit of blood transfusion practice in critically ill patients
Source: Crit Care. 2018 Apr 19;22:102. doi: 10.1186/s13054-018-2018-9 (PMC5909204; doi:10.1186/s13054-018-2018-9)
Supplement: Supplementary file 2 — Tables S1-S6 and Figures S1-S3. (PDF 459 kb) [file 13054_2018_2018_MOESM2_ESM.pdf]

Worldwide audit of blood transfusion practice in critically ill patients

Jean-Louis Vincent et al on behalf of the ICON Investigators

ADDITIONAL FILE 2

Table S1. Patients' characteristics, outcome, and RBC transfusion according to geographic region

	**Africa**	**Middle East**	**South America**	**North America**	**West Europe**	**East Europe**	**Oceania**	**East & Southeast Asia**	**South Asia**
**Characteristic**									
Age, mean ± SD	48.1±18.9	55.1±20.6	58.4±20.2	58.7±18.4	62.6±17.1	60.3±16.9	58.3±18.0	59.3±18.2	54.7±17.0
Sex, male, n (%)	81 (62.8)	207 (56.4)	468 (52.7)	384 (55.2)	2469 (61.1)	616 (56.9)	260 (59.8)	532 (60.0)	652 (71.0)
Any comorbidity, n (%)	37 (28.5)	181 (49.1)	393 (43.6)	386 (55.0)	1968 (48.2)	527 (48.3)	180 (41.0)	372 (41.5)	314 (33.5)
Severity scores on admission to the ICU, mean ± SD									
SAPS II score	38.0±16.6	43.9±20.1	42.9±18.0	36.6±16.4	43.1±16.4	43.1±17.5	41.4±18.1	44.3±16.6	31.7±16.7
SOFA score	6.4±4.4	7.4±4.3	6.0±4.2	5.9±4.7	6.6±4.1	6.3±4.2	7.0±3.8	7.4±4.4	4.3±3.8
Sepsis in the ICU	38 (29.2)	151 (40.9)	303 (33.6)	147 (20.6)	1357 (33.2)	336 (30.8)	135 (30.8)	372 (41.5)	134 (14.3)
Mortality rates, n (%)									
In-hospital	24 (20.9)	126 (36.1)	231 (29.6)	92 (13.2)	890 (22.6)	284 (27.0)	60 (13.8)	189 (22.8)	110 (13.9)
ICU	22 (17.3)	101 (27.9)	186 (22.0)	66 (9.4)	624 (15.5)	235 (21.7)	45 (10.3)	137 (15.8)	91 (10.6)
Length of stay, days, median (IQ)									
Hospital	8 (3-18)	11 (5-24)	9 (5-20)	6 (3-14)	12 (6-22)	10 (6-18)	8 (4-17)	11 (5-25)	6 (2-10)
ICU	2 (1-5)	4 (2-9)	4 (2-7)	2 (1-4)	3 (1-7)	3 (2-7)	2 (1-5)	4 (2-7)	2 (1-4)
Blood transfusion, n (%)									
On ICU admission	24 (18.5)	37 (10.0)	87 (9.7)	99 (14.1)	515 (12.6)	212 (19.4)	40 (9.1)	111 (12.4)	67 (7.1)
Overall	38 (29.2)	114 (30.9)	211 (23.4)	190 (27.1)	1105 (27.0)	381 (34.9)	92 (21.0)	239 (26.7)	141 (15.0)

Missing values: age, sex, and type of admission in 50, 114, and 479 patients, respectively; percentages are calculated taking into account missing values
ICU: intensive care unit; SAPS: simplified acute physiology score; SOFA: sequential organ failure assessment; SD: standard deviation; IQ: interquartile range

Figure S1. Hemoglobin concentrations over the first 2 weeks following admission to the ICU in patients who received a blood transfusion (right panel) and those who did not (left panel). The minimum (triangles) and maximum (circles) hemoglobin values recorded daily are displayed as means with 95% confidence interval (CI)

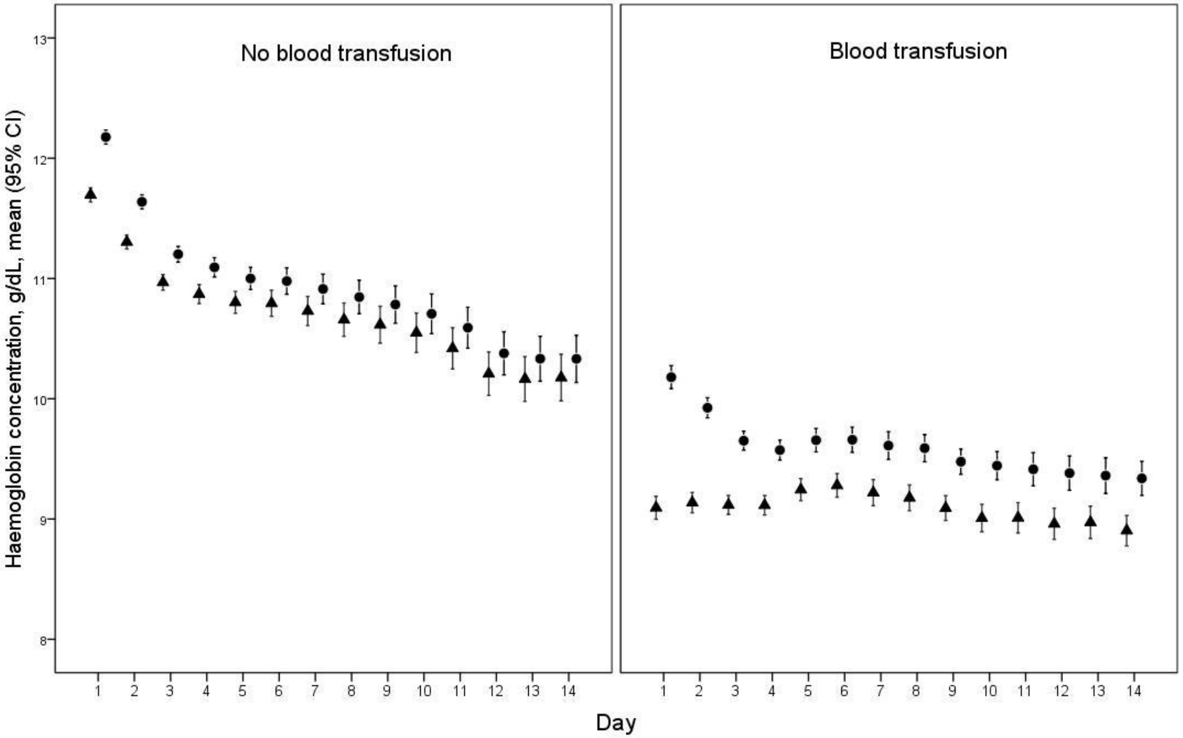

Table S2. Severity scores on admission, mortality rates, length of stay and SOFA scores during the ICU stay according to the minimum hemoglobin concentration on admission and during the ICU stay.

Hb categories, g/dl	n=9553*	Severity scores on ICU admission, mean ± SD		Mortality, n (%)		Length of stay, median (IQR)		SOFA score during ICU stay, mean ± SD	
		SAPS II	SOFA-Score	ICU	Hospital	ICU	Hospital	SOFA max	SOFA mean
On admission									
< 7	407	49.4 ± 20.0§	8.8 ± 4.9§	118 (30.3)§	146 (38.6)§	3 (1-7)§	9 (3-21)§	10.7 ± 5.2§	8.6 ± 4.7§
7-8.9	2055	44.1 ± 17.2	7.4 ± 4.3	354 (17.6)	498 (25.6)	4 (2-8)	12 (6-25)	9.2 ± 4.8	6.9 ± 4.1
9-10.9	2953	41.2 ± 16.9	6.6 ± 4.1	457 (15.8)	616 (22.0)	3 (2-7)	11 (6-22)	8.3 ± 4.5	6.1 ± 3.7
11-13	2333	39.8 ± 17.4	5.7 ± 4.0	311 (13.7)	414 (18.9)	3 (1-6)	9 (4-16)	7.2 ± 4.4	5.3 ± 3.6
> 13	1588	37.4 ± 17.9	5.2 ± 4.1	225 (14.6)	282 (18.9)	2 (1-5)	7 (3-14)	6.7 ± 4.5	5.0 ± 3.7
During the ICU stay									
< 7	1169	48.0 ± 18.1§	8.6 ± 4.5§	316 (27.6)§	414 (37.3)§	6 (3-15)§	15 (7-32)§	11.2 ± 5.0§	8.1 ± 4.3§
7-8.9	2953	43.7 ± 16.7	7.3 ± 4.1	508 (17.2)	699 (24.8)	4 (2-9)	13 (7-26)	9.2 ± 6.7	6.7 ± 3.9
9-10.9	2443	40.5 ± 16.9	6.0 ± 3.9	308 (12.9)	415 (18.1)	3 (2-5)	10 (5-18)	7.4 ± 4.1	5.5 ± 3.5
11-13	1701	38.4 ± 17.9	5.3 ± 3.9	205 (12.4)	268 (16.8)	2 (1-4)	7 (3-13)	6.5 ± 4.2	5.0 ± 3.7
> 13	1070	34.8 ± 17.6	4.5 ± 3.8	128 (12.3)	160 (16.0)	2 (1-3)	5 (2-10)	5.7 ± 4.1	4.5 ± 3.6

*missing Hb values: n=217.

§ $p<0.001$ among groups. Hb - hemoglobin; IQ - interquartile; ICU – intensive care unit; n – number of patients ; SAPS II - Simplified Acute Physiology Score II; SD - Standard- deviation; SOFA - Sequential Organ Failure Assessment

Figure S2. Relationship between the nadir hemoglobin concentration on the day of ICU admission, the sequential organ failure assessment (SOFA) score and ICU and hospital mortality

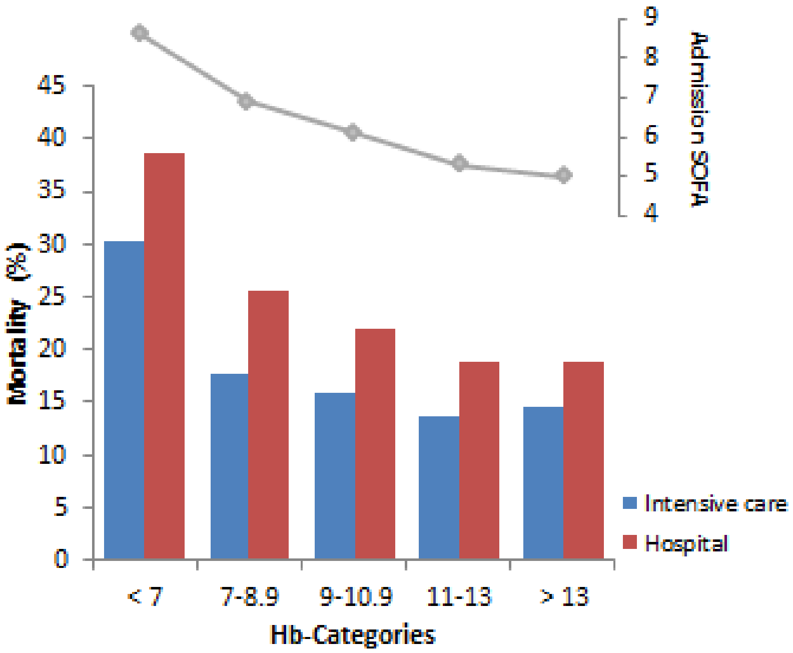

Table S3. Blood transfusion according to the minimum hemoglobin concentration at any time during the ICU stay and mortality rates according to the transfusion status

	Hemoglobin concentration, g/dL*											
	<7		7.1-8		8.1-9		9.1-10		10.1-13		>13	
	RBC transfusion, n (%)		RBC transfusion, n (%)		RBC transfusion, n (%)		RBC transfusion, n (%)		RBC transfusion, n (%)		RBC transfusion, n (%)	
**Outcome**	No	Yes	No	Yes	No	Yes	No	Yes	No	Yes	No	Yes
	293 (38)	478 (62)	616 (45.3)	744 (54.7)	1010 (61.7)	626 (38.3)	1157 (78.7)	314 (21.3)	2699 (92.3)	226 (7.7)	1052 (96.3)	40 (3.7)
Mortality rate, %†												
ICU	28.1	27.0	18.4	21.2	15.1	20.0 ‡	13.2	17.3	12.2	23.3 ‡	12.3	12.8
Hospital	36.6	37.7	24.5	30.8	21.4	27.3 ‡	18.3	22.8	6.9	25.2 ‡	15.8	32.4‡

Missing values: Hemoglobin concentration (n=298), ICU mortality (n=218), hospital mortality (n=528).
* The minimum reported hemoglobin concentration during the ICU stay in patients who did not receive blood transfusions. In patients who received blood transfusions the mean transfusion trigger (minimum hemoglobin concentration on the day of transfusion) was considered.
† Valid percentage after exclusion of the missing values.
‡ p-value <0.05 vs. no transfusion.

Table S4. Rate of transfusion according to ICU length of stay

ICU length of stay, days	N	Transfusion during the ICU stay, n (%)
≤ 1	2197	244 (11.1)
2	1734	302 (17.4)
3 - 4	2026	522 (25.8)
5 - 8	1665	511 (30.7)
≥9.00	1612	857 (53.2)

Figure S3. Hemoglobin concentration prior to blood transfusion on admission or at any time during the ICU stay according to SAPS II (upper panel) and SOFA (lower panel) scores

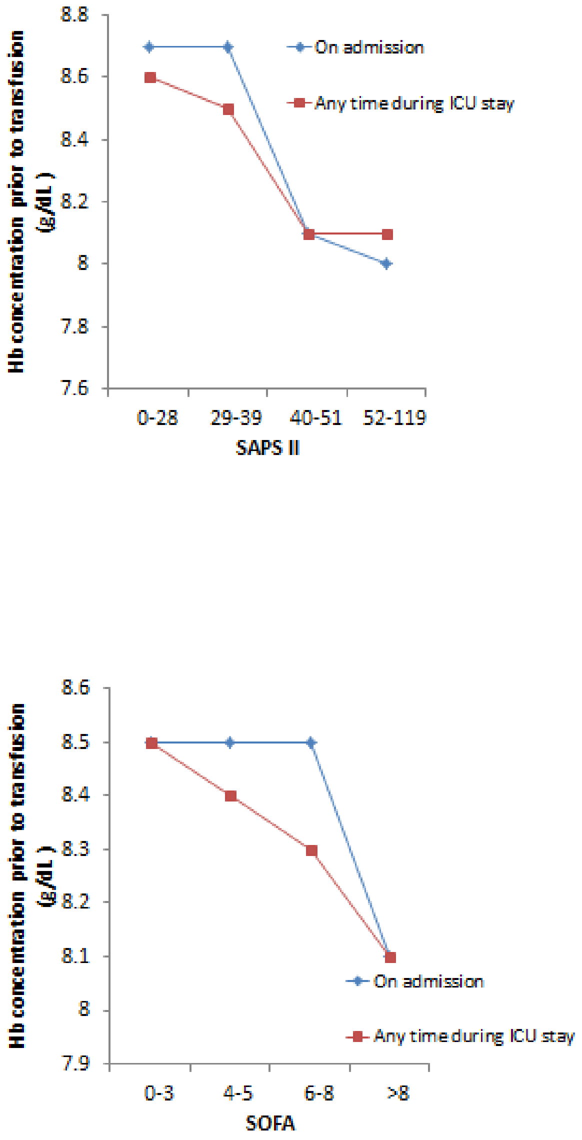

Table S5. Mortality and morbidity according to transfusion status and the number of transfused red blood cell units

		Severity score on the day of ICU admission, mean±SD		Mortality, n (%)		Length of stay, median (IQR)		SOFA score during ICU stay, mean±SD	
	n	SAPS II	SOFA	ICU	Hospital	ICU	Hospital	SOFA max	SOFA mean
Transfusion on day of ICU admission									
No	8361	40.7 ± 17.6	6.1 ± 4.1	1269 (15.6)	1676 (21.4)	3 (2-6)	9.5 (5-20)	7.8 ± 4.6	5.8 ± 3.8
Yes	1192	45.3 ± 18.3	8.1 ± 4.5	238 (20.3)	330 (29.2)	4 (2-7)	11 (6-22)	9.8 ± 5.0	7.4 ± 4.3
Transfusion during ICU stay									
No	7042	39.7 ± 17.7	5.7 ± 4.0	976 (14.3)	1286 (19.6)	3 (1-5)	8 (4-16)	7.2 ± 4.4	5.4 ± 3.7
Yes	2511	45.7 ± 17.1	8.1 ± 4.3	531 (21.5)	720 (30.0)	5 (3-12)	15 (7-30)	10.4 ± 4.9	7.5 ± 4.1
Number of transfused RBC units									
On admission to the ICU									
0	8361	40.7 ± 17.6§	6.1 ± 4.1§	1269 (15.6)§	1676 (21.4)§	3 (2-6)§	9.5 (5-20)§	7.8 ± 4.6§	5.8 ± 3.8§
1	350	43.9 ± 17.1	7.5 ± 4.3	52 (15.1)	86 (25.5)	4 (2-7)	12 (7-24.5)	9.1 ± 4.9	6.9 ± 4.0
2	446	44.2 ± 18.2	7.8 ± 4.5	86 (19.5)	122 (29.0)	4 (2-7)	11 (6-22)	9.6 ± 5.1	7.2 ± 4.3
3	153	45.3 ± 18.1	8.1 ± 4.4	32 (21.6)	42 (29.0)	3 (2-6)	10.5 (5.3-18.8)	9.8 ± 5.0	7.4 ± 4.2
4	113	48.6 ± 19.0	8.8 ± 4.5	27 (24.3)	35 (32.7)	4 (2-6)	10 (5.8-21.5)	10.4 ± 4.9	8.1 ± 4.4
≥5	130	49.5 ± 20.3	10.1 ± 5.1	41 (32.3)	45 (36.6)	3 (1-7)	10 (2-21)	11.3 ± 5.2	9.2 ± 5.1
At any time during ICU stay									
0	7042	39.7 ± 17.7§	5.7 ± 4.0§	976 (14.3)§	1286 (19.6)§	3 (1-5)§	8 (4-16)§	7.2 ± 4.4§	5.4 ± 3.7§
1	499	43.0 ± 15.3	7.0 ± 3.9	70 (14.2)	103 (21.4)	4 (2-8)	13 (7-25)	8.7 ± 4.4	6.4 ± 3.7
2	724	44.7 ± 17.1	7.7 ± 4.2	135 (19.1)	193 (28.2)	5 (2-10)	14 (7-26)	9.5 ± 4.6	7.0 ± 4.0
3	307	45.9 ± 16.8	8.0 ± 4.0	65 (21.5)	85 (28.7)	5 (2-9)	13 (7-23.8)	10.2 ± 4.6	7.4 ± 3.8
4	287	47.0 ± 18.1	8.4 ± 4.0	58 (20.6)	80 (29.1)	5 (3-12)	14 (7-30)	10.7 ± 4.6	7.7 ± 3.9
≥5	694	47.9 ± 17.8	9.2 ± 4.6	203 (29.7)	259 (38.8)	10 (4-20)	20 (10-39)	12.4 ± 5.0	8.7 ± 4.4

RBC- red blood cell; IQR – Interquartile; ICU – intensive care unit; n – number of patients; SAPS II - Simplified Acute Physiology Score II; SD - standard deviation; SOFA - Sequential Organ Failure Assessment

§ p <0.001 among groups

Table S6. Summary of univariate and multivariable analyses with hospital mortality as the dependent variable

**Characteristic**	**Univariate analysis**		**Multivariable analysis***	
	Hazard ratio (95% CI)	p-value	Hazard ratio (95% CI)	p-value
Age (per year)	1.02 [1.02-1.02]	<0.001	1.01 [1.00-1.01]	0.004
Male	0.94 [0.86-1.02]	0.148	0.97 [0.88-1.08]	0.593
SAPS II (per point)	1.06 [1.06-1.06]	<0.001	1.04 [1.04-1.05]	<0.001
SOFA scores				
SOFAresp	1.47 [1.42-1.53]	<0.001	1.11 [1.06-1.17]	<0.001
SOFAcoag	1.24 [1.19-1.29]	<0.001	1.10 [1.05-1.16]	<0.001
SOFAhep	1.15 [1.11-1.18]	<0.001	1.02 [0.98-1.07]	0.333
SOFAcns	1.52 [1.48-1.56]	<0.001	1.07 [1.03-1.12]	0.002
SOFAren	2.45 [2.24-2.69]	<0.001	1.14 [1.10-1.19]	<0.001
SOFAcvs	1.39 [1.35-1.43]	<0.001	1.06 [1.02-1.10]	<0.001
Comorbidities				
COPD	1.23 [1.09-1.40]	<0.001	0.90 [0.78-1.04]	0.169
Cancer. non-metastatic	1.27 [1.11-1.45]	<0.001	1.43 [1.22-1.68]	<0.001
Cancer. metastatic	1.73 [1.42-2.10]	<0.001	1.39 [1.08-1.78]	0.011
Hematologic cancer	2.54 [2.06-3.12]	0.326	0.99 [0.75-1.29]	0.915
Diabetes mellitus	1.07 [0.93-1.24]	<0.001	1.03 [0.87-1.21]	0.759
Heart failure (NYHA III-IV)	1.91 [1.69-2.16]	<0.001	1.16 [1.00-1.34]	0.055
Renal failure	1.60 [1.40-1.82]	<0.001	0.97 [0.82-1.13]	0.669
HIV	1.98 [1.33-2.94]	<0.001	0.86 [0.56-1.32]	0.488
Cirrhosis	2.15 [1.79-2.57]	<0.001	1.48 [1.19-1.83]	<0.001
Immunosuppression	1.57 [1.36-1.80]	<0.001	1.09 [0.92-1.30]	0.304
MV on ICU admission	2.65 [2.41-2.92]	<0.001	1.29 [1.12-1.48]	<0.001
RRT on ICU admission	2.12 [1.83-2.45]	<0.001	0.67 [0.55-0.82]	<0.001
Sepsis on ICU admission	2.17 [1.98-2.39]	<0.001	1.03 [0.92-1.16]	0.589
Hemoglobin concentration on admission, g/dl				
<7	R	NA		

7-9	0.57 [0.48-0.69]	<0.001	0.82 [0.66-1.01]	0.063
9-11	0.48 [0.40-0.58]	<0.001	0.75 [0.61-0.93]	0.009
11-13	0.42 [0.35-0.50]	<0.001	0.76 [0.61-0.95]	0.015
>13	0.42 [0.35-0.52]	<0.001	0.88 [0.69-1.11]	0.277
Type of admission				
Surgical	R	NA		
Medical	1.83 [1.65-2.02]	<0.001	1.59 [1.40-1.80]	<0.001
Trauma	1.04 [0.84-1.30]	0.702	1.86 [1.42-2.42]	<0.001
Others	2.14 [1.32-3.47]	0.002	2.71 [1.62-4.51]	<0.001
RBC transfusion†	1.04 [1.02-1.05]	<0.001	0.98 [0.96-1.00]	0.048

*Cox proportional hazard analysis with in-hospital death as the dependent variable in the whole population adjusted for geographic region, hospital and ICU organizational characteristics (type of hospital, hospital bed capacity, nurse to patient ratio and the presence of an ICU specialist 24 h/day)

† introduced as a time-dependent variable in relation to the time to the first RBC unit transfused.

SOFA: sequential organ failure assessment; COPD: chronic obstructive pulmonary disease; RRT: renal replacement therapy; RBC: red blood cell; MV: mechanical ventilation
